# Semiconductor Chip Electrical Interconnection and Bonding by Nano-Locking with Ultra-Fine Bond-Line Thickness

**DOI:** 10.3390/nano11081901

**Published:** 2021-07-24

**Authors:** Jielin Guo, Yu-Chou Shih, Roozbeh Sheikhi, Jiun-Pyng You, Frank G. Shi

**Affiliations:** 1 Department of Materials and Manufacturing Technology, Henry Samueli School of Engineering, University of California, Irvine, CA 92617, USA; rsheikhi@uci.edu; 2 Department of Chemical and Biomolecular Engineering, Henry Samueli School of Engineering, University of California, Irvine, CA 92617, USA; yuchous@uci.edu (Y.-C.S.); fgshi@uci.edu (F.G.S.); 3 Hangzhou GOL Nanotechnology LTD, Hangzhou 310053, China; jpyou20004@yahoo.com

**Keywords:** nanoscale locking (NL), bond-line thickness, heterogenous integration, electrical contact resistance, flip-chip LED, junction temperature, wet high temperature operating life (WHTOL)

## Abstract

The potential of an innovation for establishing a simultaneous mechanical, thermal, and electrical connection between two metallic surfaces without requiring a prior time-consuming and expensive surface nanoscopic planarization and without requiring any intermediate conductive material has been explored. The method takes advantage of the intrinsic nanoscopic surface roughness on the interconnecting surfaces: the two surfaces are locked together for electrical interconnection and bonding with a conventional die bonder, and the connection is stabilized by a dielectric adhesive filled into nanoscale valleys on the interconnecting surfaces. This “nano-locking” (NL) method for chip interconnection and bonding is demonstrated by its application for the attachment of high-power GaN-based semiconductor dies to its device substrate. The bond-line thickness of the present NL method achieved is under 100 nm and several hundred times thinner than those achieved using mainstream bonding methods, resulting in a lower overall device thermal resistance and reduced electrical resistance, and thus an improved overall device performance and reliability. Different bond-line thickness strongly influences the overall contact area between the bonding surfaces, and in turn results in different contact resistance of the packaged devices enabled by the NL method and therefore changes the device performance and reliability. The present work opens a new direction for scalable, reliable, and simple nanoscale off-chip electrical interconnection and bonding for nano- and micro-electrical devices. Besides, the present method applies to the bonding of any surfaces with intrinsic or engineered surface nanoscopic structures as well.

## 1. Introduction

System-on-chips, including chiplets, enabled by 3D or 2.5D integration of semiconductor dies from separate or the same wafers onto a single chip, represent a new paradigm for advantages beyond Moore’s law [1]. This new paradigm is critically dependent on the continuing innovation for die-to-die, die-to-wafer, die-to-interposer, die-to-substrate or board, die-to-redistribution layer, and wafer-to-wafer, interpose-to-substrate electrical interconnection and bonding [2]. Mainstream off-chip electrical interconnection and bonding methods include the bump-based approach using electrically conductive bumps, assisted by thermocompression or thermosonic tools. Copper pillars based advanced bump has the smallest interconnect pitch being limited to 40 μm, i.e., a 25 μm Cu bump with 15 μm spacing. To further shrink the Cu bump pitch, a strict surface nanoscale flatness is necessary [3]. The second mainstream method is the bumpless approach which eliminates the need for intermediate conductive bump and a much reduced interconnect pitch down to 2 μm or lower becomes possible, if the roughness of the interconnecting surfaces is reduced to below 0.1 nm via CMP (chemical mechanical polishing). In some cases, the surfaces must undergo an additional pre-annealing plasma activation in an ultra-high vacuum [4].

Although the interconnection pitch has a paramount significance for enhancing the device integration density, it is noted that the ultimate goal of advancing integration schemes is to reduce the device power consumption and to increase the device speed with a lower cost. Thus, in addition to the lateral interconnection pitch, the vertical dimension or thickness of the interconnection is similarly critical. For 2.5D and 3D ICs, through silicon via (TSV) interposer [5], hybrid bonding [6], and embedded multi-die interconnect bridge technology (EMIB) [7] are all developed for vertically stacked chips in order to increase the integrated density while lower the signal travel distance and reduce the power consumption. For example, with a 3D die stack, the issue of hot spot within the stack due to an enhanced thermal resistance contributes to its much slower commercial adoption than interposer-based 2.5D integration with a less sever thermal issue [8]. It has been very difficult to enhance the off-chip EI performance by following the on-chip size scaling approach. To achieve the necessary industry-standard reliability, the bond-line thickness (BLT) for the EI based on typical DAA or solder is often required to be as thick as over 25 or 30 μm, respectively, while for the EI using DAF, the BLT is still as thick as 10 μm. Recent innovative efforts on off-chip EIs include the method of MesoGlue [9], nanoscale-surface-sculpturing [10], nanosized Ag powder sintering [11], nanoporous (NP) metals [12], and nano- and micro-metal pastes [13], however, unfortunately, none of them is demonstrated to be able to reduce the BLT to below one micrometer. The size reduction in off-chip EIs often suffers from insurmountable electrical and reliability issues [14].

The significance of the vertical interconnection dimension or bond-line thickness (BLT) is even more evident in the case of die-to-substrate bonding in power semiconductor devices based on wide band gap (WBG) SiC and GaN, which are being explored as another route to extend Moore’s law and to replace silicon for many needed emerging applications [1,2]. However, the apparent potential of WBG semiconductors is currently far from being realized because of lacking a more advanced die interconnection and bonding method for achieving a much lower electrical (and thermal) resistance than current methods including the much-explored nano-silver sintering bonding [15].

Thus, in addition to the continuing overall push for an ever-shrinking lateral pitch size, there are compelling reasons to seek innovative methods for reducing the vertical bond-line thickness significantly of off-chip electrical interconnections than what can be achieved by the current mainstream bump or bumpless method. Thus, in our earlier published work, a new method named “Nano-locking” (NL) takes advantage of the intrinsic nanoscopic surface roughness on the interconnecting surfaces has been proposed: the two surfaces are locked together for electrical interconnection and bonding with a conventional die bonder, and the connection is stabilized by a dielectric adhesive filled into nanoscale valleys on the interconnecting surfaces [16]. The electrical and thermal conduction of the “nano-locking” bonding method is built through the contacts between the ridges and valleys on the roughness of the two bonding surfaces. The surface morphology plays an important role in determining the contact resistance between the two bonding surfaces. Without adding any metallic fillers, the vertical dimension of the bonding can be controlled as thin as nanometer scale and the adhesion strength can also be largely improved by reducing the risk of delamination at the interface between the dielectric adhesive and the bonding surfaces.

The objective of the present work reports the further development of our NL method into the regime of nanoscale interconnection with a bond-line thickness (BLT) less than 100 nm and the influence of BLT on the performance evaluation of the packaged devices enabled by the present NL method is shown. This NL method for chip interconnection and bonding is demonstrated by its application for the attachment of high-power GaN-based light-emitting diode (LED) dies to its device substrate. The BLT achieved can be shown to be as low as 30 nm, several hundred times thinner than those achieved using mainstream bonding methods, resulting in a lower overall device thermal resistance, and a reduced electrical resistance, and thus an improved overall device performance and reliability. Different BLT results in different overall contact area between the two bonding surfaces and therefore leads to different contact resistance and affect the optical, thermal performances, as well as the reliability.

## 2. Materials and Methods

### 2.1. Nano-Locking (NL) Electrical Interconnection Method

**Figure 1:**
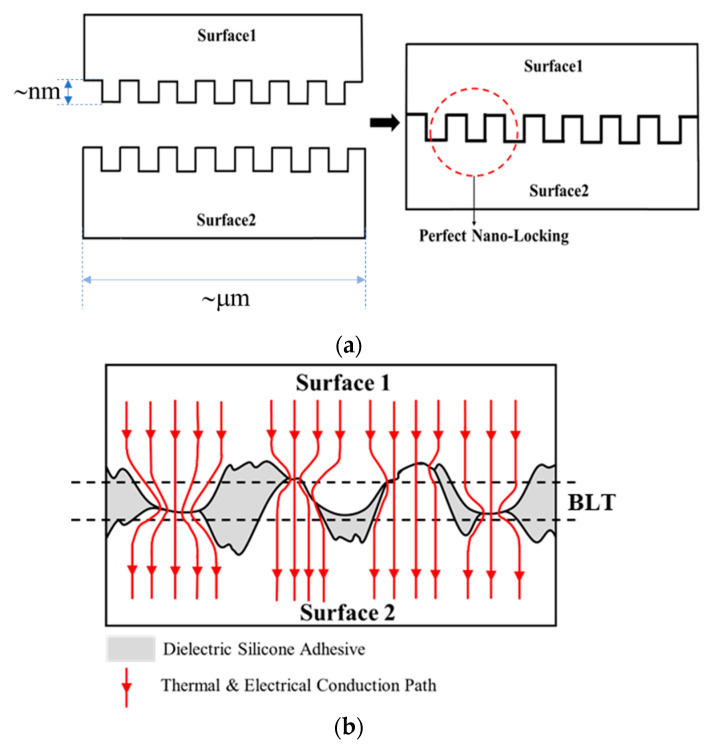
Schematic illustration of the nano-locking (NL) method for chip interconnection and bonding: (**a**) a perfect locking for two surfaces with exact matching structures and (**b**) a locking for general interconnecting surfaces with random intrinsic structures: electrical and thermal interconnections are established (the indicated pathways) when the surfaces are in contact, and the interconnection is stabilized and the two surfaces are bonded by the adhesive filled in the surface valleys (the gray area).

Figure 1 illustrates general aspects of the present NL method: Figure 1a shows the interconnection and bonding between two surfaces with exact matching structures without any intermediate material. In general, however, the intrinsic surface structures are not as exactly matching as in Figure 1a, but they are random as shown in Figure 1b. The electrical interconnection of such two metallic surfaces can be established when the nanoscopic structures from the interconnecting surfaces are brought to be in contact, and such an interconnection can be mechanically stabilized and by filling the nanoscopic valleys with a structural adhesive, as illustrated in Figure 1b.

**Figure 2:**
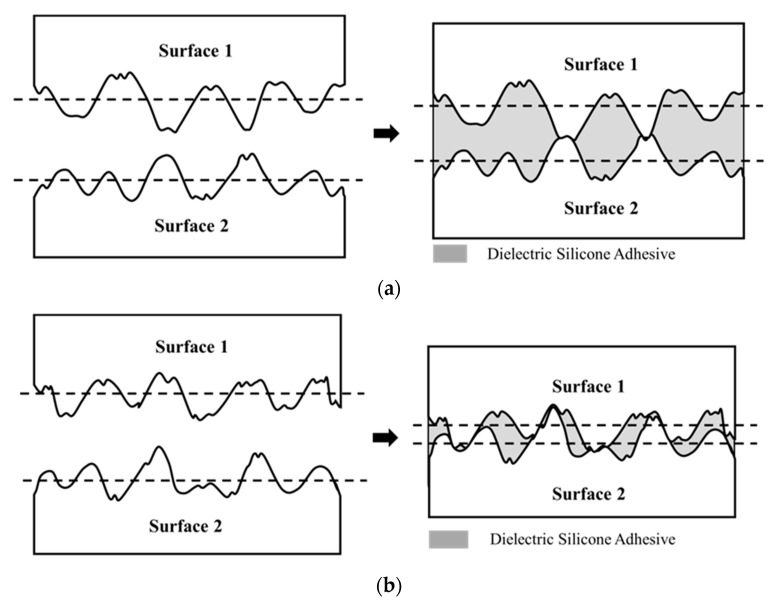
Schematic illustration of the BLT limits: (**a**) the maximum BLT and (**b**) the minimum BLT. The maximum BLT is achieved when the two highest ridges on two surfaces are contact with each other, and the minimum BLT is reached when the highest ridge on surface 2 is contact with the deepest valley on surface 1.

The bond-line thickness is defined as the vertical distance between the baseline of the surface roughness on the two bonding surfaces as shown in Figure 1b. The potential range of BLT is within the maximum and minimum limits, controlled by the highest ridges and deepest valleys on the interconnecting surfaces, as illustrated by Figure 2a,b, while the specific BLT value (within the maximum and minimum range) obtained during a die bonding process is dependent on the bonding pressure.

The robustness of the NL method is demonstrated by bonding high-power GaN based dies to the device substrate, i.e., the bonding between the metallic pad on the semiconductor die and the pad on the package substrate, without using any intermediate conductive material, and without requiring a pre-bonding surface planarization. The as-received commercially available semiconductor dies and packages are employed in the present work. The dies used are flip-chip type light emitting diodes (LEDs) with the size of 1 × 1 mm^2^ and a forward voltage of 3.0 V (San’an Optoelectronics Corp.). The specified maximum operating DC current is 700 mA with an emission peak at 455 nm. The composition of die pads consists of Ti/Ni/Au. The package substrate has a size of 5 × 6 mm^2^ and consists of an optically reflective cup and the heatsink slug (Jufei Optoelectronics Corp.). The composition of substrate pad is Cu plated with Ag.

### 2.2. Fabrication of Packaged Devices

The fabrication of semiconductor device packaging process includes die bonding, curing, encapsulation, and soldered the encapsulated device to an Al-based printed circuit board (PCB) as described in our earlier manuscript [17]. For the purpose of comparison, the devices made by using conventional die–substrate bonding methods are prepared, i.e., one is bonded by using a commercial silver-epoxy adhesive with a silver flake amount of 85% by weight, and its volume resistivity is 8 × 10^−5^ Ωm, and another is bonded by using AuSn (80% gold and 20% tin) eutectic solder with a volume resistivity of 1.64 × 10^−7^ Ωm. The associated BLT is based on the industrial standard for reliability, i.e., 25 ± 2 μm for the silver-epoxy bonding and 20 ± 2 μm for the AuSn eutectic bonding, respectively [18].

### 2.3. Devices Performance Evaluation

The surface roughness of those metallic pads is determined by atomic force microscopy (Anton Paar Tosca 400 AFM, Graz, Austria) using an Arrow NCR cantilever with a reflective aluminum coating which has a typical tip radius of < 10 nm, resonance frequency of 285 kHz, and spring constant of 42 N/m. Images were acquired using a scan rate of 1 line/s and measurement region of 50 × 50 μm^2^. The current–voltage (I-V) behavior of the fabricated device is determined by using the Keithley 2450 source meter (Cleveland, OH, USA). The junction temperature and thermal resistance for the overall die–substrate interconnection and bonding layer is measured under natural convection condition [19]. The current source with corresponding forward voltage is supplied by Everfine power generator (Hangzhou, China), and the lumen output is measured in a LabSphere integral sphere (North Sutton, NH, USA) with the suggested maximum input constant current of 700 mA. The wet high temperature operation life (WHTOL) reliability test goes beyond the requirement of the industrial standard JEDEC No.22-A101C by extending the test duration by 25% from 1000 h to 1250 h. All the samples are placed on the heat sink and evaluated by measuring the lumen maintenance as a function of aging time at a temperature of 85 °C and a relative humidity of 85% with the maximum suggested input DC current of 700 mA, which is aged in a high temperature and humidity chamber (GLMP50, Chemkorea Corp. (GLMP50, Chemkorea Corp., Irvine, CA, USA)).

## 3. Results and Discussion

### 3.1. Pad Surface Morphology

**Figure 3:**
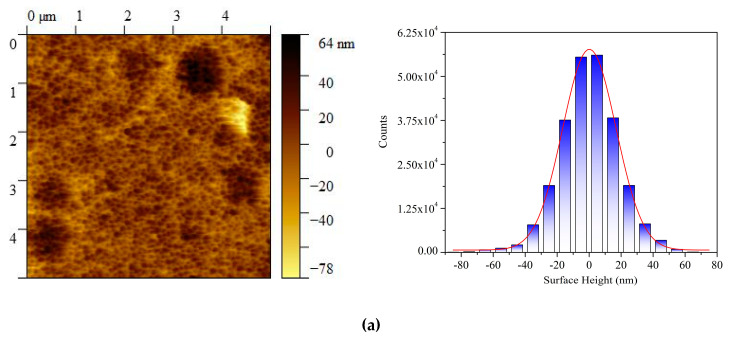

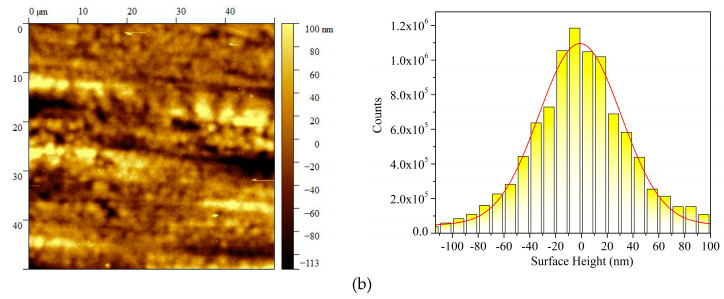
AFM images for the surface topography and surface roughness distribution (**a**) the semiconductor die metallic pad and (**b**) the package substrate metallic pad.

Figure 3 presents surface morphology for the die and substrate metallic pads. As shown in Figure 3a, the AFM image of the die pad shows surface topography and the histogram of roughness height distribution. The surface roughness ranges from −78 ± 2 nm to +64 ± 2 nm. Figure 3b presents the AFM image as well as the surface roughness distribution of the substrate pad. It is noted that the surface roughness ranges from −113 ± 2 nm to +100 ± 2 nm. As a result, the maximum BLT which equals the sum of the highest ridges (on both die and substrate metallic pads) as illustrated in Figure 2a, is (100 ± 2) + (64 ± 2) = 164 ± 4 nm. Similarly, the minimum BLT which equals the value of highest ridge (on substrate metallic pad) minus the value of deepest valley (on die metallic pad), as illustrated in Figure 2b, is (100 ± 2) − (78 ± 2) = 22 ± 4 nm. Therefore, the possible BLT range using the present NL approach is between 22 ± 4 and 164 ± 4 nm. Indeed, three sets of devices made using the present NL approach have the respective BLT value of 28 ± 5 nm, 47 ± 5 nm, and 85 ± 5 nm, which are evidently within the BLT range by controlling the applied pressure during the die bonding process.

### 3.2. Devices Performance: Electrical

**Figure 4:**
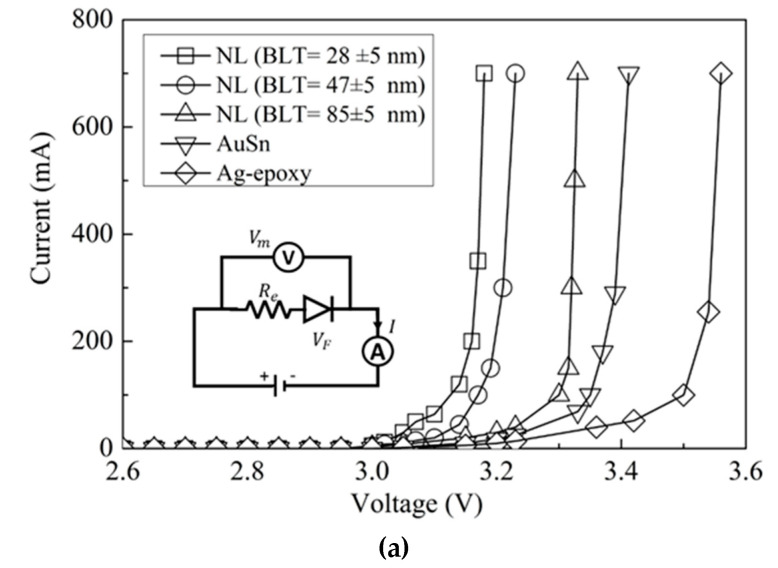

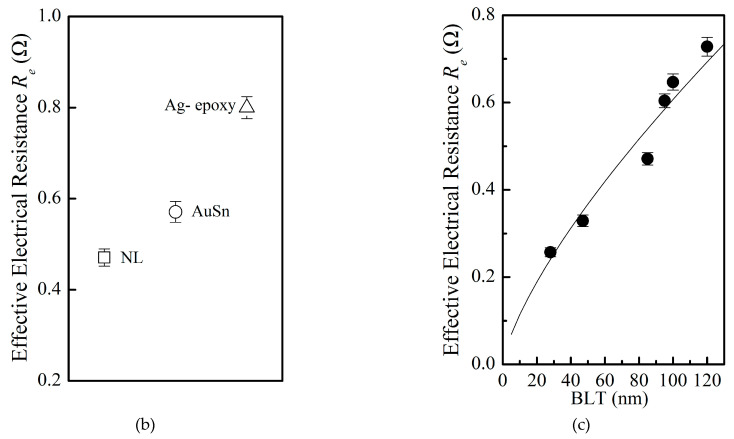
(**a**) Measurement of current (I) and voltage (V) relationship for the devices made of the present NL and conventional die–substrate interconnection and bonding methods. The open square symbol (□) represents the NL bonding with BLT = 28 ± 5 nm, the open circle symbol (○) represents the NL bonding with BLT = 47 ± 5 nm, the open triangle symbol (Δ) represents the NL bonding with BLT = 85 ± 5 nm, the open triangle symbol (∇) represents the AuSn bonding; the open rhombus symbol (◊) represents the Ag-epoxy bonding. The solid curve represents the best I-V fitting. (**b**) The $R_{e}$ of different die–substrate bonding and connection methods: the open square symbol (□) represents NL bonding with BLT = 85 ± 5 nm, the open circle symbol (○) represents AuSn bonding, the open triangle symbol (Δ) represents Ag-epoxy bonding. (**c**) The solid circle symbol (●) correspond to the extracted values for $R_{e}$ in the NL approach, which indicates $R_{e}$ decreases with decreasing BLT which can be described by $R_{e}\;$ = 0.0215${\;(\mathrm{BLT})}^{0.7254\;}$ (the solid curve).

Figure 4 presents the electrical performance for devices made with the NL method, and its comparison to two conventional die-substrate bonding methods. According to Figure 4a, the measured voltage for the devices made by the NL approach is less than that for the devices using other two conventional methods, under the same forward current of 700 mA. Thus, a reduced interconnection electrical resistance is evident for the devices made by the present NL approach, which is supported by the result presented in Figure 4b for the effective interconnection electrical resistance $R_{e}\;$= dV/dI = ($V_{m}-V_{f}{)/I}_{f}$, where $V_{m}$ is the measured device voltage, $V_{f}$ is the forward voltage, and $I_{f}$ is the forward current. Figure 4b presents the extracted $R_{e}$ of different die-substrate bonding methods: the $R_{e}$ for the devices made by the NL approach with a BLT of 85 ± 5 nm is ~12% lower than the conventional AuSn bonding method and is about 35% lower than the conventional Ag-epoxy bonding.

Furthermore, as shown in Figure 4c, for the devices made by the present NL approach, $R_{e}$ decreases significantly with decreasing BLT, and such a BLT dependence of $R_{e}$ follows a relationship of $R_{e}\;$= 0.0215${\;(\mathrm{BLT})}^{0.7254}.$ This is expected in view of the prior result on the relationship between the contact resistance and applied bonding pressure [20], and in the present case the BLT is inversely dependent on the applied pressure.

In addition to the nanometer scale BLT achieved for the first time with the present NL method, another significant advantage is the present interconnection is not critically affected by any possible interfacial defects resulting from voids and delamination formed during die bonding process associated with the two conventional methods. This type of interfacial poor contact increases the interfacial electrical and thermal resistance and degrades fatally the corresponding electrical and thermal performance and reliability [21]. In the present NL method, however, the only function of the adhesive used is for mechanically bonding, and it does not impact on the electrical and thermal resistance of the formed interconnection, so long a reasonable bonding is achieved, even with some defects. The electrical and thermal performance in the present method is more strongly related to the individual contact area of nano-contacts on the interconnecting surfaces, and the total number of those contacts, which is thus strongly dependent on the BLT: a reduced BLT results in a larger individual contact area, as well as an increased number of contacts [22].

### 3.3. Device Performance: Thermal

**Figure 5:**
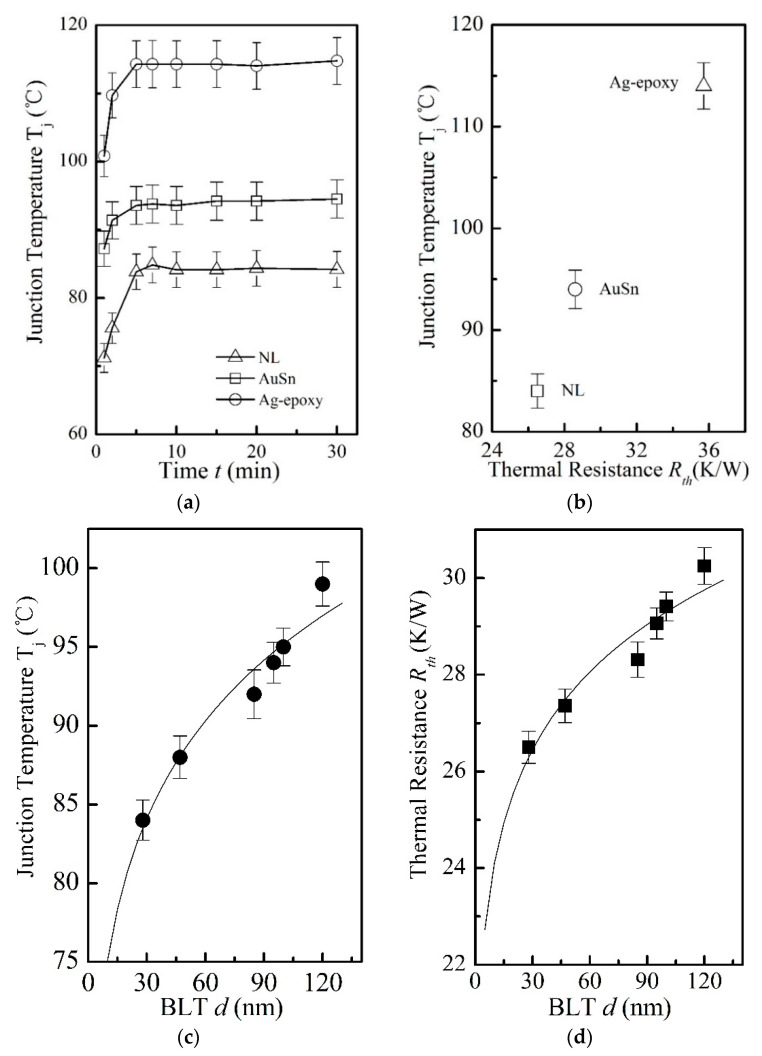
(**a**) The die junction temperature ($T_{j}$) of the devices made by three different die–substrate bonding methods: the open triangle symbol (Δ) represents the experimental measurement of $T_{j}$ for the device made with the NL approach with a BLT of 28 ± 5 nm. The open square symbol (□) represents $T_{j}$ data for the device made by the AuSn bonding with an industrial standard BLT value of 20 ± 2 μm, the open circle symbol (○) represents $T_{j}$ data for the device made w with Ag-epoxy bonding with an industrial standard BLT value of 25 ± 2 μm. The solid lines represent the best fitting. (**b**) The relationship between $T_{j}$ and $R_{th}$: the open square symbol (□) represents NL bonding with BLT = 28 ± 5 nm, the open circle symbol (○) represents AuSn bonding, the open triangle symbol (Δ) represents Ag-epoxy bonding. (**c**) For the devices made by the NL bonding, $T_{j}\;$ is found to decrease with the decreasing BLT, which can be described by $T_{j}$ = 59.311 ${\left(\mathrm{BLT}\right)}^{0.1027}$ ( the solid curve), and the solid circle symbol (●) represents the experimental data for $T_{j}$; (**d**) For the device made by the NL bonding, $R_{\mathrm{th}}$ is found to decrease with the decreasing BLT, as described by $R_{th}$ = 19.826${\;(\mathrm{BLT})}^{0.0848\;}$ (the solid curve). The solid square symbol (■) represents the extracted thermal resistance $R_{\mathrm{th}}$.

Figure 5 presents the thermal performance for the devices made with the present NL approach and two conventional die–substrate bonding methods. Figure 5a presents the measurement of the die junction temperature ($T_{j})$. It is evident from the device made by the NL method with a BLT = 28 ± 5 nm, a much lower $T_{j}$ is resulted, which is ~10 °C lower than the AuSn bonding, and ~30 °C lower than Ag-epoxy bonding. This significant difference in $T_{j}$ is also reflected in the huge difference in the corresponding thermal resistance of the die–substrate bonding layer made with different die bonding methods as shown by Figure 5b: the thermal resistance ${(R}_{\mathrm{th}})$ for the NL bonding with a BLT = 28 ± 5 nm, is about 7% lower than the AuSn bonding, and about 26% lower than the Ag-epoxy die bonding.

In addition, for the devices made with the present NL approach, $T_{j}$ decreases with decreasing BLT, and the BLT dependence of $T_{j}$ follows $T_{j}\;$= 59.311 ${\left(\mathrm{BLT}\right)}^{0.1027}$ as shown in Figure 5c. Similarly, $R_{\mathrm{th}}$ decreases with decreasing BLT, and the BLT dependence of $R_{\mathrm{th}}$ follows $R_{\mathrm{th}}\;$= 19.826 ${\left(\mathrm{BLT}\right)}^{0.0848\;}$ as shown in Figure 5d, which is fully understandable in view of the prior result on the dependence of thermal resistance on the thickness of bonding layer [23]. As the value of BLT depends on the bonding pressure, and thus the $T_{j}$ and $R_{\mathrm{th}}$ are essentially a dependence on the bonding pressure.

As discussed above, the relatively significant large thermal resistance values associated with Ag-epoxy and AuSn bonding, might to do with avoidable imperfect die-substrate interfacial bonding resulting from voids and other defects during bonding process.

### 3.4. Device Performance: Optical

**Figure 6:**
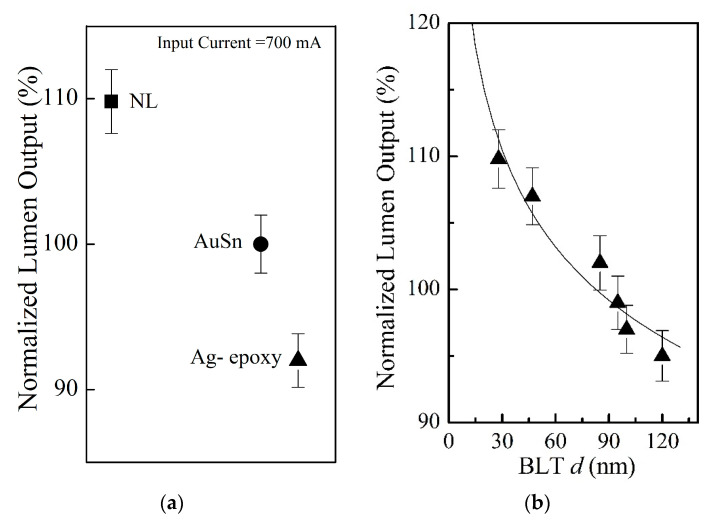
(**a**) Normalized lumen output of the devices made by three different die-substrate bonding methods at an input current of 700 mA: the solid square symbol (■) represents the NL bonding approach with BLT = 28 ± 5 nm, the solid circle symbol (●) represents AuSn bonding with an industrial standard BLT value of 20 ± 2 μm, the solid triangle symbol (▲) represents Ag-epoxy bonding with an industrial standard BLT value of 25 ± 2 μm. (**b**) For the devices made by the NL bonding, the normalized lumen output ($I_{m}$) is found to be increase with the decreasing BLT, which can be described by $I_{m}=1.5414{\;(\mathrm{BLT})}^{-0.098}$ (the solid curve), and the solid triangle symbol (▲) represents the experimental data for normalized lumen output ($I_{m}$ ).

Figure 6a presents the optical performance in terms of normalized lumen output at the suggested maximum input current of 700 mA for the devices made with the NL method, and the comparison to two conventional die–substrate bonding methods. It is evident that the devices made with the NL method (BLT = 28 ± 5 nm) results in a much higher lumen output *(I_m_):* approximately 9.8% higher compared with the AuSn bonding, and about 17% higher compared with the Ag-epoxy bonding. For present NL method, lumen output *(I_m_)* decreases with increasing BLT and the BLT dependence of lumen output ($I_{m}$) follows $I_{m}=1{.5414\;(\mathrm{BLT})}^{-0.098}$ as shown in Figure 6b. This is fully consistent with the prior results on the dependence of $T_{j}$ and $R_{\mathrm{th}}$ on the applied pressure since BLT is inversely proportional to the applied pressure. Therefore, the normalized lumen output dependence on BLT in Figure 6b is essentially dependent on the bonding pressure.

### 3.5. Device Performance: Reliability

**Figure 7:**
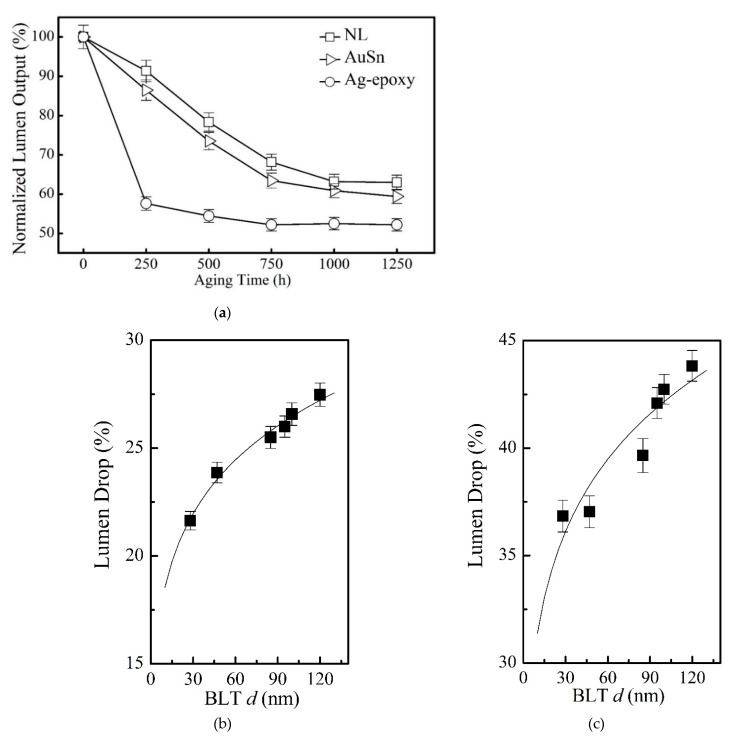
(**a**) Long-term lumen maintenance of the devices with three different die–substrate bonding methods as a function of aging time under the stressing condition of an operating current of 700 mA, a relative humidity RH = 85%, and a high environmental temperature of 85 °C: the solid square symbol (□) represents the experimental data of NL bonding with BLT = 28 ± 5 nm, the open triangle symbol (▷) represents the experimental data of AuSn bonding, the open circle symbol (○) represents the experimental data of Ag-epoxy bonding. This Wet High Temperature Operating Life (WHTOL) test goes beyond the requirement of the standard JEDEC No.22-A101C while extending the test duration by 25% from 1000 to 1250 hr. (**b**) The lumen drop ($I_{d}$) at aging time of 500 hr vs BLT(d): it follows a power law relationship $I_{d}=0{.1298d}^{0.1547}$ as presented by the solid line; (**c**) The lumen drop ($I_{d}$ ) at aging time of 1000 hr vs BLT: it follows a power law relationship $I_{d}=0{.2336d}^{0.1282}$ as presented by the solid line.

Figure 7a presents a comparison of the aging time-dependent lumen maintenance of the devices made with the NL approach and the conventional methods under the industrial standard condition of high chamber temperature of 85 °C and high relative humidity of 85% for a total duration of 1250 hr. The y-axis represents relative change in the lumen maintenance normalized to the initial lumen output. The x-axis represents the aging time. It is evidently that at the aging time of 1250 hr, the NL method in the case of BLT = 28 ± 5 nm results in a much higher lumen output, i.e., about 4% higher than device made by the AuSn bonding method, and ~13% higher than the device made by the Ag-epoxy bonding method. The superior reliability associated with the NL approach is evidently resulted from the observed reduced electrical resistance as well as a reduction in thermal resistance, as discussed above.

Figure 7b,c describes the lumen drop ($I_{d}$) at different aging time of packaged devices with different BLTs. The dependence of lumen-drop ($I_{d}$) on BLT (d) at an aging time of 500 hr follows a power law relationship $I_{d\;}=0{.1298d}^{0.1547}$ as shown in Figure 7b. The same happens to the dependence of lumen-drop ($I_{d}$) on BLT (d) at an aging time of 1000 hr follows a power law relationship $I_{d}=0{.2336d}^{0.1282}$ as shown in Figure 7c. This is fully consistent with the prior result on the dependence of lumen output on the applied pressure as BLT is inversely proportional to the applied pressure.

## 4. Conclusions

The potential of an innovation for establishing a simultaneous mechanical, thermal, and electrical connection between two metallic surfaces without requiring a prior time-consuming and expensive surface nanoscopic planarization and without requiring any intermediate conductive material has been explored. The “nanoscale-locking” bonding method takes advantage of the intrinsic nanoscopic surface roughness on the interconnecting surfaces: the two surfaces are locked together for electrical interconnection and bonding with a conventional die bonder, and the connection is stabilized by a dielectric adhesive filled into nanoscale valleys on the interconnecting surfaces. This NL method for chip interconnection and bonding has been demonstrated as an example by its application for the attachment of high-power GaN-based semiconductor dies to its device substrate. The bond-line thickness achieved has been shown to be as low as 30 nm, several hundred times thinner than those achieved using mainstream bonding methods, resulting in a lower overall device thermal resistance, and a reduced electrical resistance, and thus an improved overall device performance and reliability. The bond-line thickness plays an important role in determining the overall contact area between the two bonding surfaces and in turn affect the contact resistance as well as device performance and long-term reliability. The present work opens a new direction for scalable, reliable and simple nanoscale off-chip electrical interconnection and bonding for nano- and micro-electrical devices as well as other functional devices. In addition, the present method applies to the bonding of any surfaces with intrinsic or engineered surface nanoscopic structures as well.
